# Supplementary material for: Revealing protonation states and tracking substrate in serine hydroxymethyltransferase with room-temperature X-ray and neutron crystallography
Source: Commun Chem. 2023 Aug 3;6:162. doi: 10.1038/s42004-023-00964-9 (PMC10397204; doi:10.1038/s42004-023-00964-9)
Supplement: Supplementary file 2 — Supplementary Information [file 42004_2023_964_MOESM2_ESM.pdf]

SUPPLEMENTAL INFORMATION

Revealing protonation states and tracking substrate in serine hydroxymethyltransferase with room-temperature X-ray and neutron crystallography

Victoria N. Drago,[1] Claudia Campos,[2] Mattea Hooper,[2] Aliyah Collins,[2] Oksana Gerlits,[2] Kevin L. Weiss,[1] Matthew P. Blakeley,[3] Robert S. Phillips,[4,5] and Andrey Kovalevsky[1]*

[1]Neutron Scattering Division, Oak Ridge National Laboratory, Oak Ridge, TN, 37831, USA
[2]Department of Natural Sciences, Tennessee Wesleyan University, Athens, TN 37303, USA
[3]Large Scale Structures Group, Institut Laue–Langevin, 71 Avenue des Martyrs, 38000 Grenoble, France
[4]Department of Chemistry, University of Georgia, Athens, GA, 30602, USA
[5]Department of Biochemistry and Molecular Biology, University of Georgia, Athens, GA, 30602, USA

* To whom correspondence should be addressed: Andrey Kovalevsky: kovalevskyay@ornl.gov

Table S1. Crystallographic data collection and refinement statistics for the joint X-ray/neutron structures of *Tth*SHMT and *Tth*SHMT/L-Ser complex. Values in parentheses are for the highest-resolution shell.

	***Tth*SHMT** PDB 8SUJ		***Tth*SHMT/L-Ser** PDB 8SUI	
**Data collection:**	**Neutron**	**X-ray**	**Neutron**	**X-ray**
Beamline/Facility	IMAGINE (HFIR, ORNL)	Rigaku HighFlux HomeLab	MaNDi (SNS, ORNL)	Rigaku HighFlux HomeLab
Space group	$P2_1$		$P2_1$	
Cell dimensions:				
a, *b*, *c* (Å); α, β, γ (°)	58.81, 83.33, 95.57; 90, 91.7, 90		58.81, 83.33, 95.57; 90, 91.7, 90	
Resolution (Å)	47.78 – 2.30 (2.42 – 2.30)	95.54 – 2.00 (2.07 – 2.00)	14.43 – 2.30 (2.38 – 2.30)	95.59 – 2.00 (2.07 – 2.00)
No. reflections measured	144559 (16968)	339198 (32160)	209756 (17169)	276584 (26180)
No. reflections unique	35286 (4490)	62364 (6226)	39816 (3920)	62552 (6227)
R_{merge}	0.161 (0.281)	0.091 (0.364)	0.228 (0.245)	0.107 (0.459)
R_{pim}	0.080 (0.145)	0.044 (0.177)	0.097 (0.115)	0.059 (0.258)
$CC_{1/2}$	0.943 (0.637)	0.993 (0.882)	0.918 (0.638)	0.991 (0.667)
$\langle I / \sigma I \rangle$	4.3 (2.9)	17.3 (3.9)	10.3 (4.7)	13.1 (2.7)
Completeness (%)	85.4 (74.7)	99.9 (99.6)	97.0 (95.9)	99.9 (99.4)
Redundancy	4.1 (3.8)	5.4 (5.2)	5.3 (4.4)	4.4 (4.2)

**Refinement:**	**Joint XN**	**Joint XN**
Resolution (neutron, Å)	40 – 2.30	40 – 2.30
Resolution (X-ray, Å)	40 – 2.00	40 – 2.00
Data rejection criteria	no observation & |F|=0	no observation & |F|=0
Sigma cut-off	2.50	2.50
No. reflections (neutron)	31065	39712
No. reflections (X-ray)	58902	58469
R_{work} / R_{free} (neutron)	0.210 / 0.239	0.204 / 0.221
R_{work} / R_{free} (X-ray)	0.164 / 0.177	0.166 / 0.183
R_{work} / R_{free} (joint XN)	0.180 / 0.199	0.181 / 0.198
No. atoms		
Protein, including H and D	12555	12557
L-Ser	N/A	14
Sulfate	10	10
Water	1287 (i.e. 429 D_2O molecules)	1188 (i,e, 396 D_2O molecules)
B-factors		
Protein	16.4	15.9
L-Ser	N/A	40.9
Sulfate	33.0	38.9
Water	38.9	38.7
R.M.S. deviations		
Bond lengths (Å)	0.008	0.007
Bond angles (°)	1.02	0.976

Table S2. Crystallographic data collection and refinement statistics for the room-temperature X-ray structure of *Tth*SHMT/D-Ser complex. Values in parentheses are for the highest-resolution shell.

	***Tth*SHMT/D-Ser** PDB ID 8SSY	**hSHMT2** PDB ID 8SSJ
**Data collection:**	**X-ray (in-house)**	**X-ray (synchrotron)**
Diffractometer	Rigaku HighFlux, Eiger R 4M	ID-19, Advanced Photon Source
Space group	$P2_1$	$P6_522$
Wavelength (Å)	1.5406	0.979
Cell dimensions:		
a, *b*, *c* (Å)	58.91, 83.47, 95.705	161.61 161.61 210.78
α, β, γ (°)	90, 91.7, 90	90, 90, 120
Resolution (Å)	100.0 – 1.80 (1.86 – 1.80)	50.0 – 2.50 (2.59-2.50)
No. reflections unique	85843 (8588)	55062 (5150)
R_{merge}	0.062 (0.322)	0.146 (0.673)
R_{pim}	0.020 (0.132)	0.063 (0.291)
$CC_{1/2}$	0.998 (0.920)	0.980 (0.765)
$<I / \sigma I>$	36.0 (4.0)	10.5 (2.0)
Completeness (%)	100.0 (100.0)	97.0 (92.7)
Redundancy	13.3 (7.2)	6.0 (5.8)
**Refinement:**		
R_{work} / R_{free}	0.1334 / 0.1645	0.1721 / 0.2046
B-factors		
Protein	20.3	41.6
D-Ser	37.8	N/A
Sulfate	47.3	61.6
Water	32.5	39.1
R.M.S. deviations		
Bond lengths (Å)	0.019	0.002
Bond angles (°)	1.597	0.522
All atom clash score	2.97	2.16

a

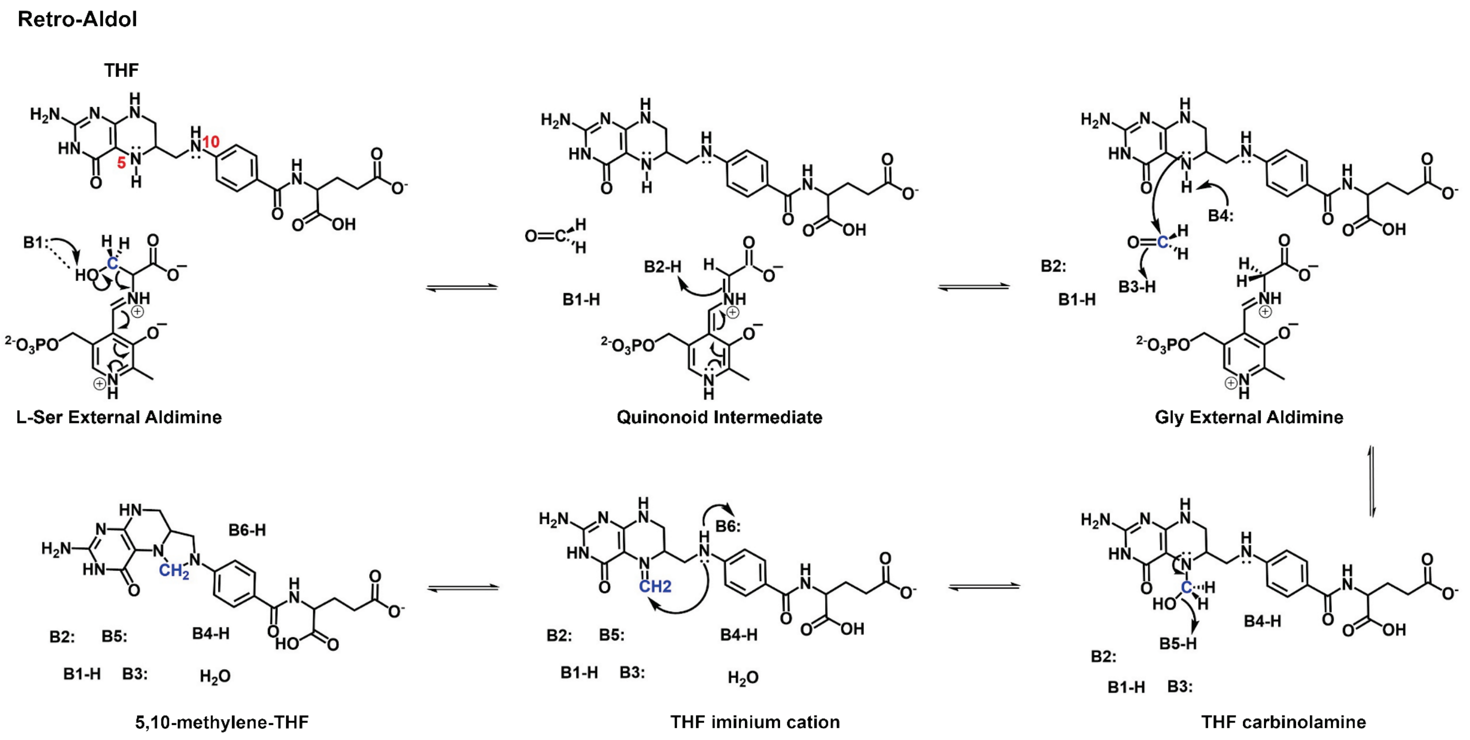
Retro-Aldol
THF
L-Ser External Aldimine
Quinonoid Intermediate
Gly External Aldimine
5,10-methylene-THF
THF iminium cation
THF carbinolamine
B1:
B1-H
B2-H
B2:
B3-H
B3:
B4:
B4-H
B5-H
B5:
B6:
B6-H
H2O

b

Concerted Mechanism

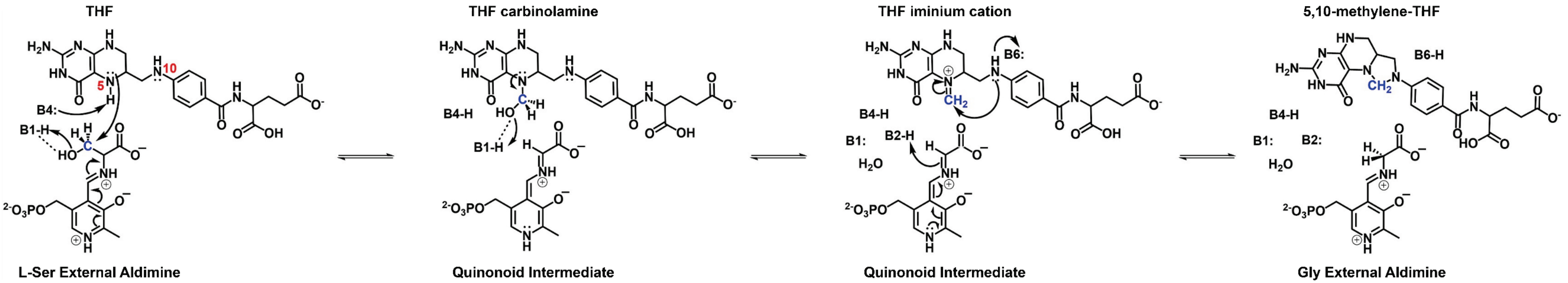

c

α-elimination

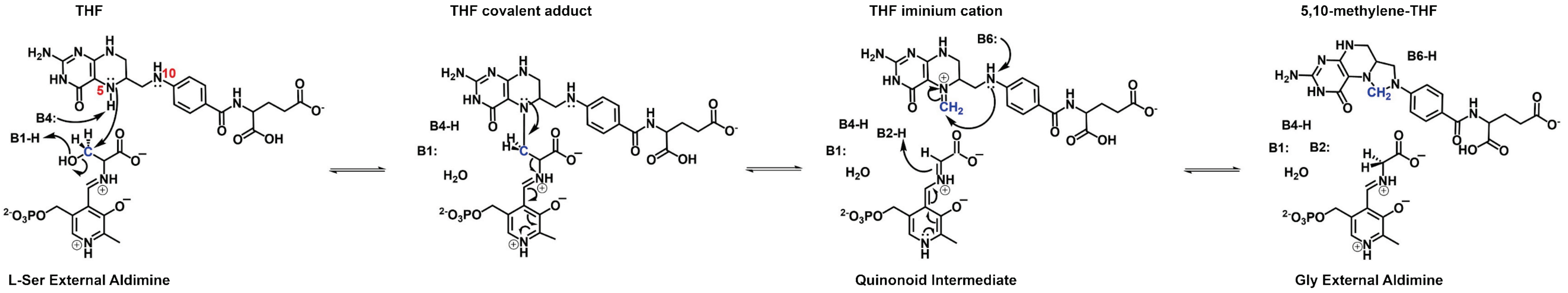

Figure S1. Predominant mechanistic hypotheses for the SHMT-catalyzed conversion of L-Ser to Gly and THF to 5,10-methylene-THF.

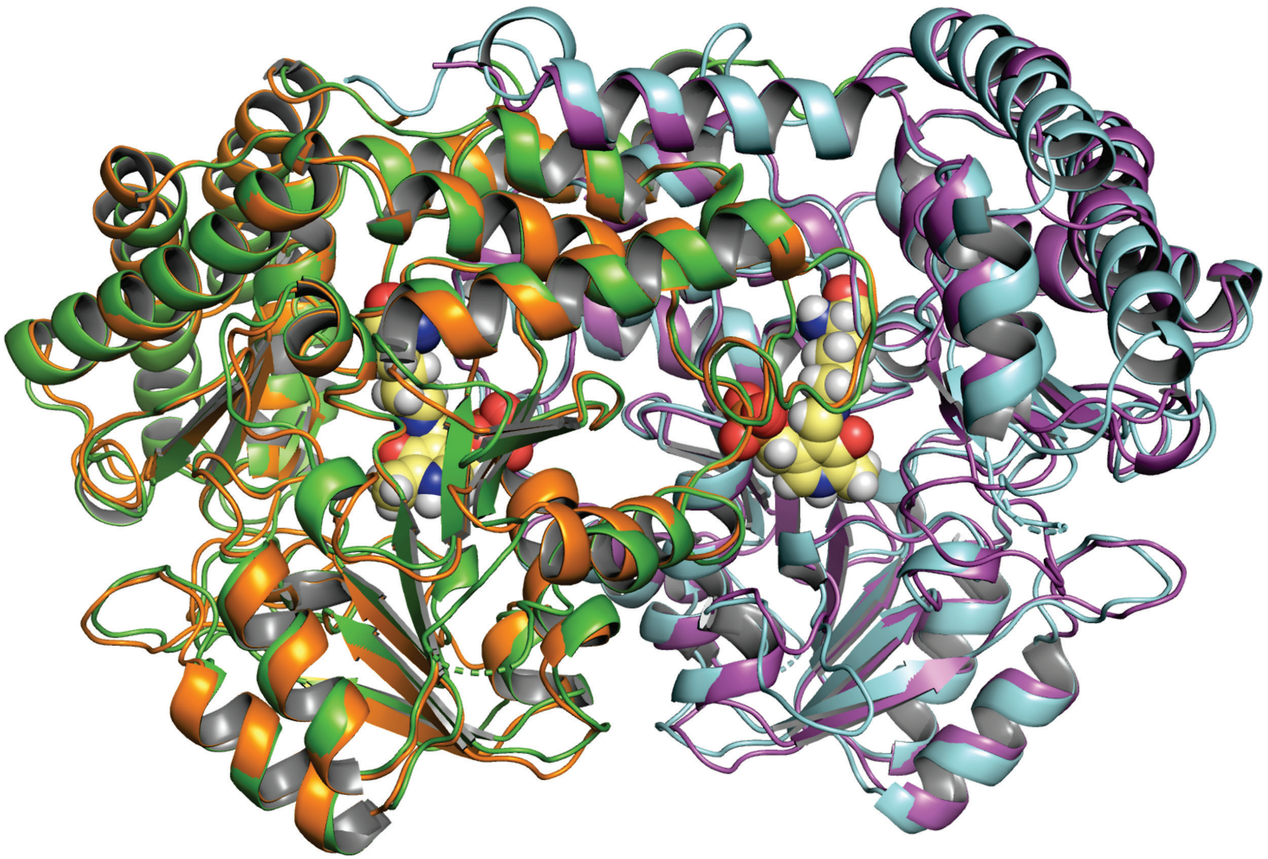

Figure S2. Superposition of *h*SHMT2 (green/cyan) and *Tth*SHMT (orange/purple) dimers. The RMSD on the main chain atoms is 0.67 Å.

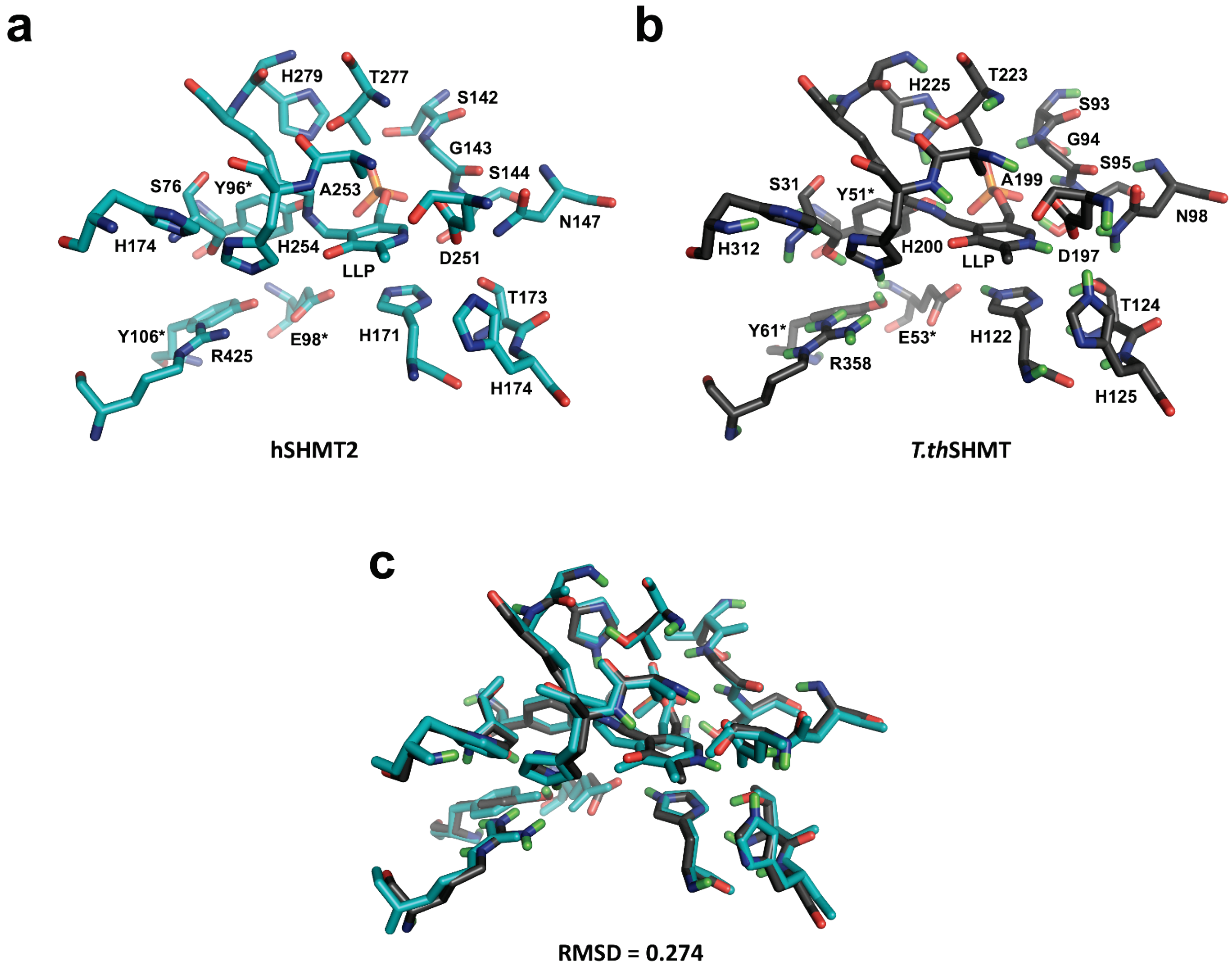

Figure S3. Comparison of conserved active sites of hSHMT2 and *Tth*SHMT.

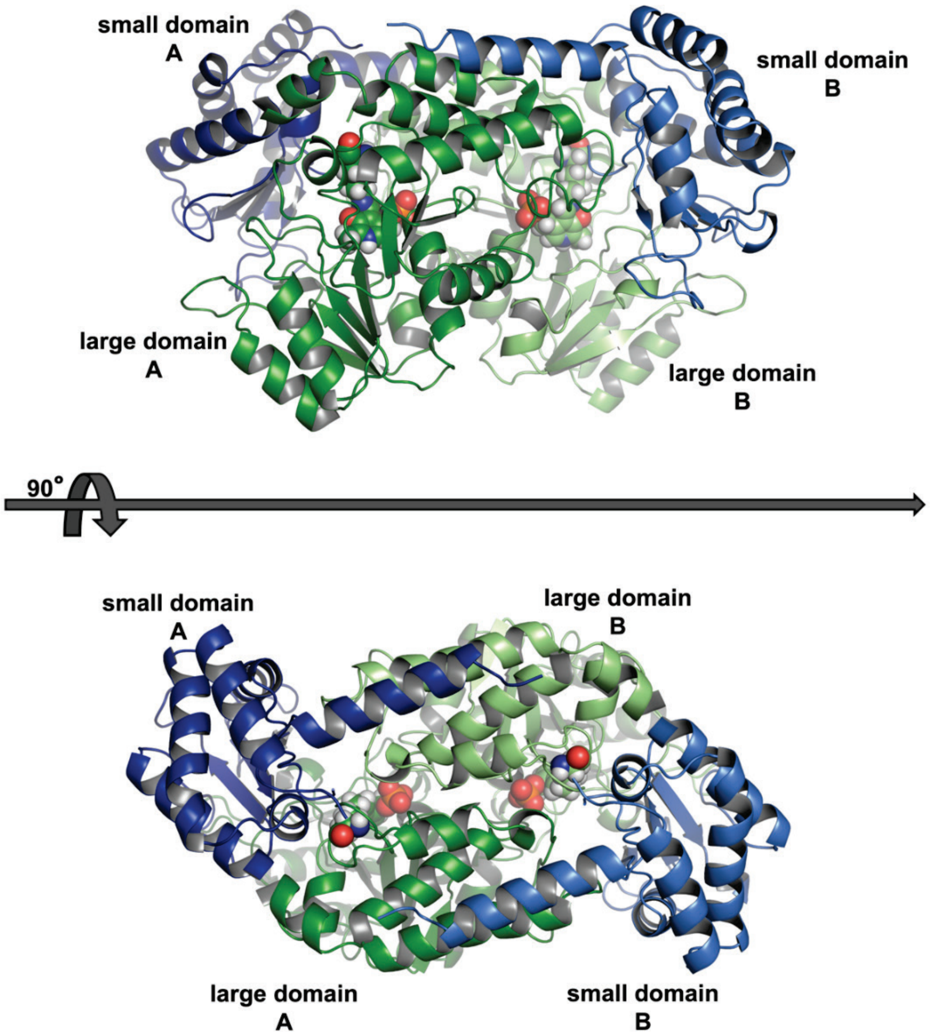

Figure S4. *Tth*SHMT dimer with small (colored blue) and large (colored green) domains labeled.

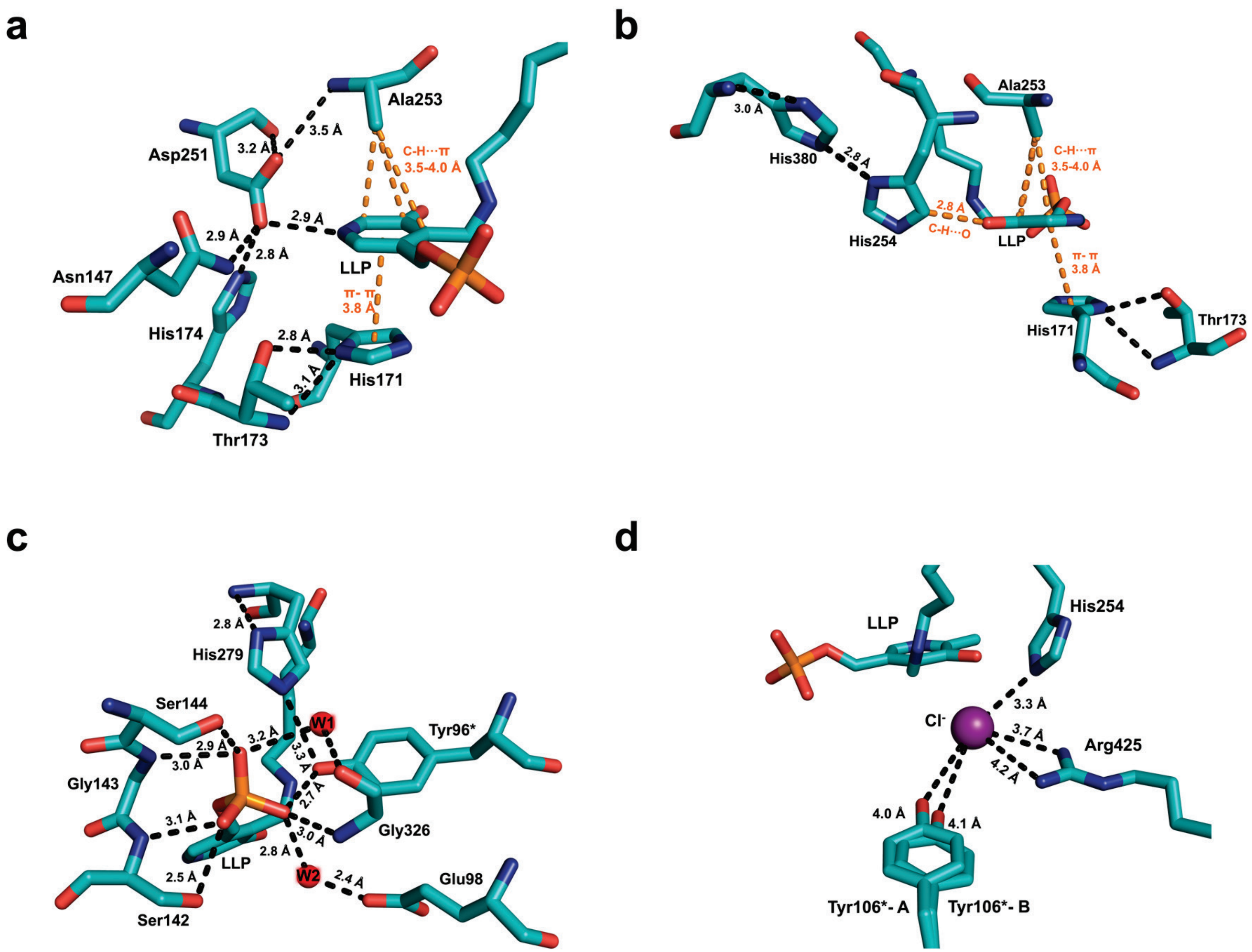

Figure S5. H-bonding and stabilizing interactions in the hSHMT2 active site in protomer B. The distances shown in Å are virtually identical in protomer A. In panel d, a chloride ion occupies the substrate binding site where a sulfate ion is observed in the structures of *Tth*SHMT.

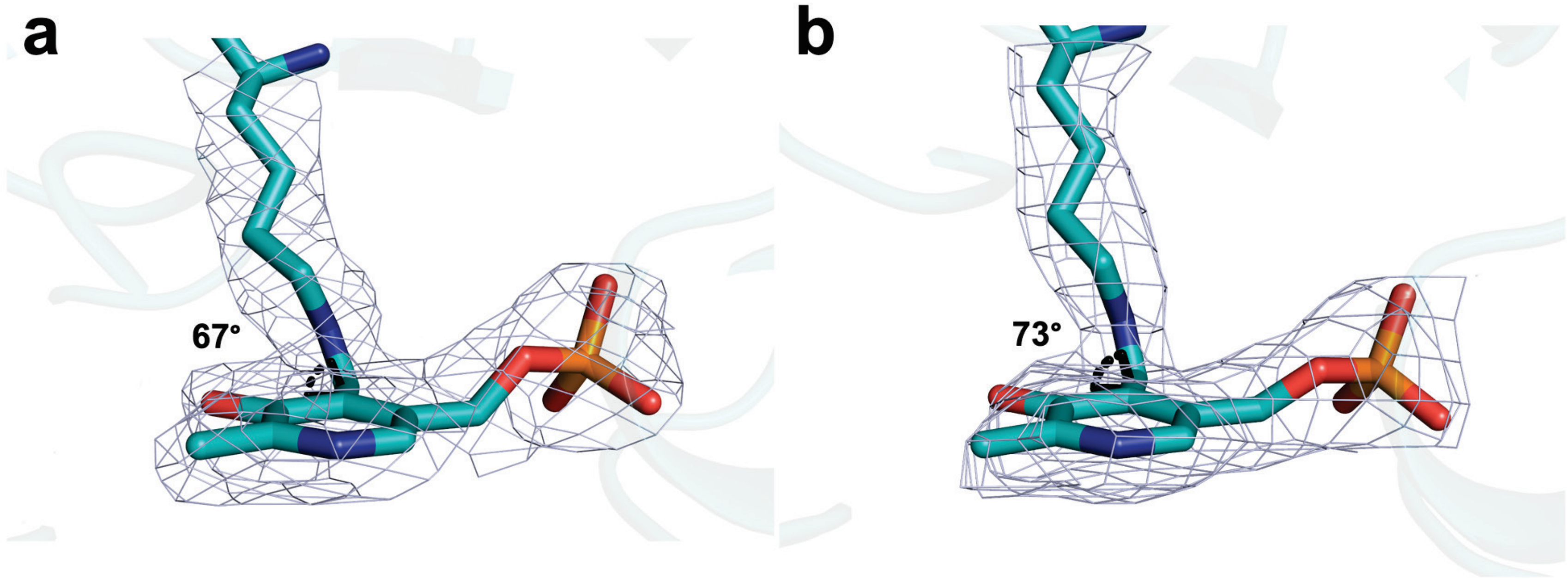

Figure S6. C3-C4-C4'-N_{SB} torsion angles observed in the PLP cofactors in **a**) protomer A and **b**) protomer B from the room-temperature X-ray structure of hSHMT2. The $2F_O$-F_C electron density contoured at 1σ level is depicted by gray-blue mesh.

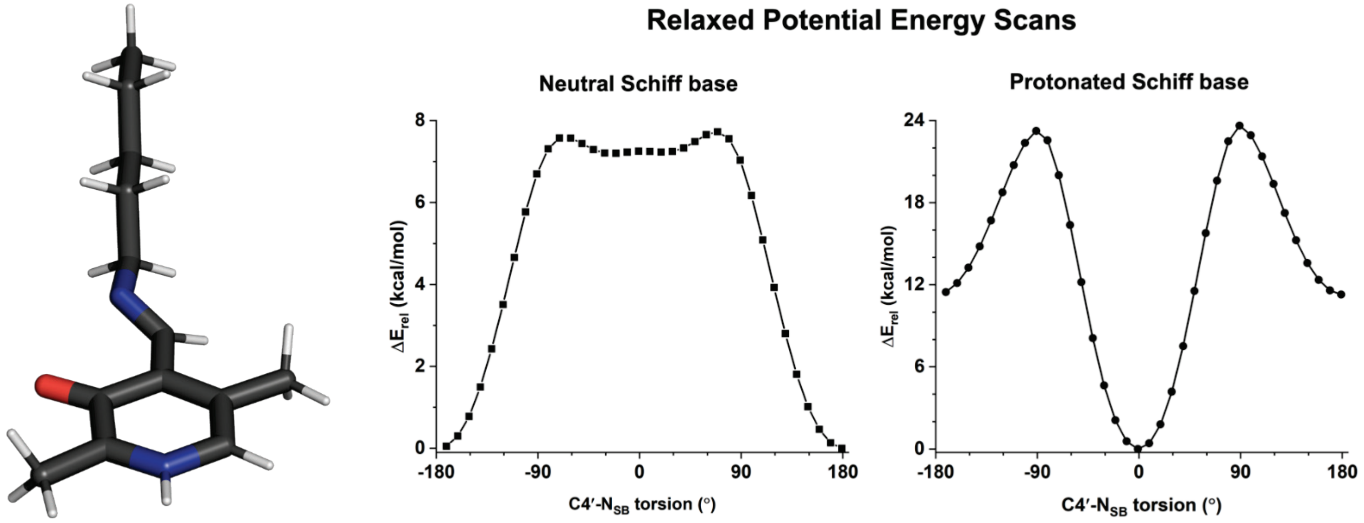

Figure S7. Internal aldimine model for the relaxed potential energy scans (left) and the potential energy profile (right) for the 360° rotation of C4′-N_{SB} bond. Energies are given in kcal mol^{-1} and are reported relative to the lowest energy state.

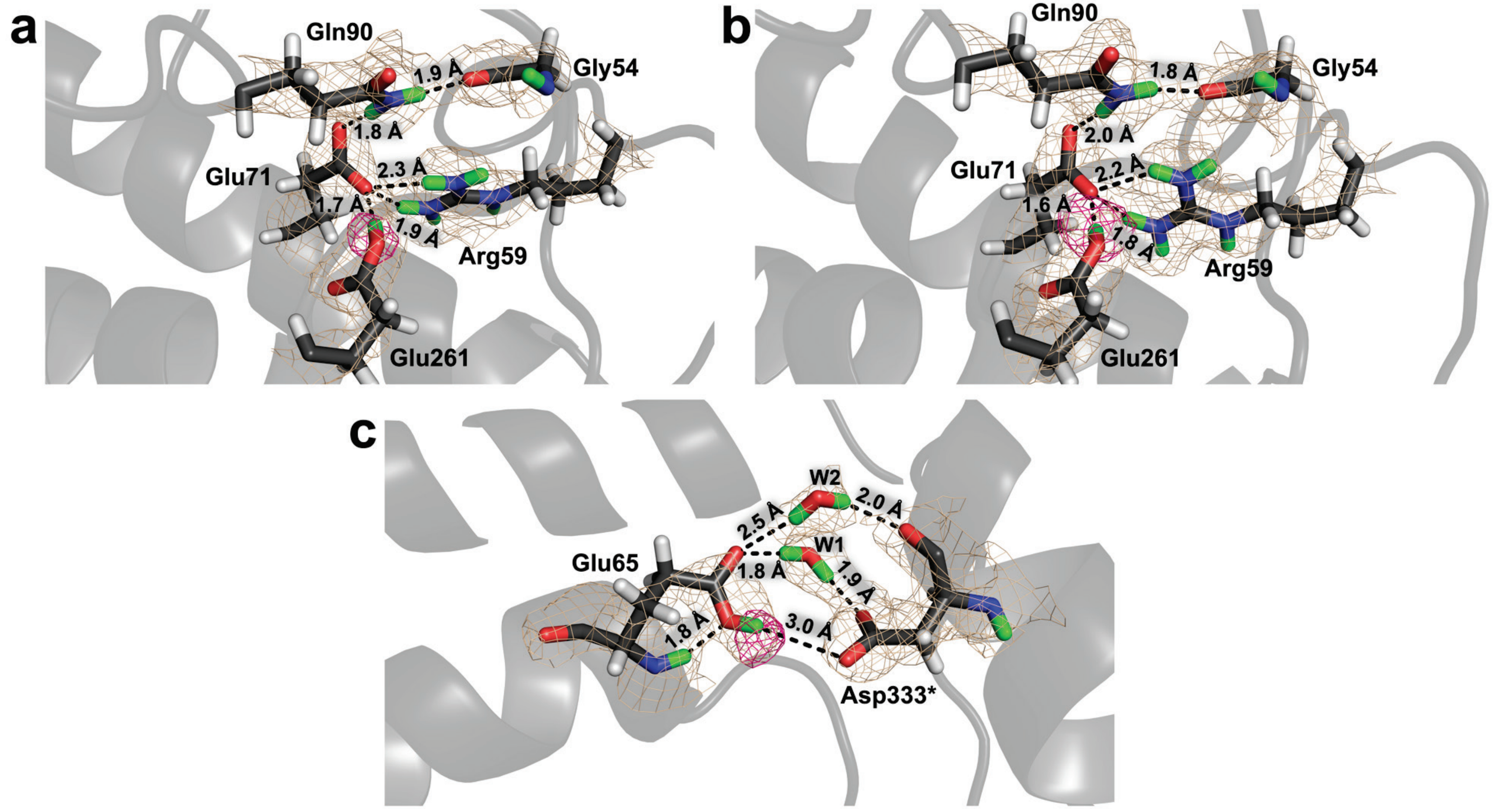

Figure S8. Protonation states of Glu residues. Glu261 (**a**) and Glu261* (**b**) in protomers A and B, respectively, are observed to be protonated and forming a H bond with Glu71 (or Glu71*) of the adjacent α-helix. Gln90, Arg59, and Gly54 assist in stabilizing this structural feature. The $2F_O$-F_C neutron scattering length density contoured at 1σ level is depicted by wheat mesh. The omit F_O-F_C difference neutron scattering length density map contoured at 3 σ level is represented by magenta mesh.

a. Codon-optimized DNA sequence.

ATGGCGCATCACCATCATCATCACGTGGGCACGGGTAGCAATGACGACGACGACAAGAGCACCAGCCTGTACAAAAAGGCTGGCAGCGC
AGCAGCACCGTTTACCGAGAATCTGTATTTCCAGAGCACTCTGAAGCGCGACGAAGCTCTTTTCGAGCTGATCGCGCTGGAAGAGAAAC
GTCAACGCGAAGGCCTGGAATTGATTGCATCCGAGAACTTTGTGTCTAAACAAGTGCGCGAGGCGGTTGGTTCCGTTCTGACGAACAAA
TATGCCGAGGGTTACCCGGGTGCACGCTATTACGGCGGCTGCGAAGTCATCGATCGTGTTGAGTCCCTGGCAATTGAGCGTGCGAAGGC
ACTGTTCGGTGCAGCGTGGGCAAACGTCCAGCCACATAGCGGCTCGCAGGCGAACATGGCCGTTTACATGGCACTGATGGAACCGGGCG
ACACCCTGATGGGCATGGATCTGGCTGCGGGTGGTCACCTGACCCACGGCAGCCGTGTCAATTTTAGCGGCAAACTGTACAAAGTCGTG
AGCTACGGCGTCCGTCCGGATACGGAATTGATCGATCTGGAAGAGGTTCGTCGCTTGGCACTGGAGCACCGTCCAAAGGTCATCGTTGC
CGGTGCTAGCGCGTATCCTCGTTTCTGGGATTTCAAAGCCTTCCGCGAAATTGCAGACGAGGTCGGTGCGTATCTGGTTGTTGATATGG
CCCATTTCGCGGGTCTGGTAGCTGCCGGTCTGCACCCAAACCCGTTGCCGTACGCGCACGTTGTGACCAGCACCACGCACAAAACGTTG
CGTGGTCCGCGTGGTGGCTTGATTCTGTCTAACGACCCGGAGCTGGGTAAGCGTATTGACAAGCTGATCTTTCCGGGTATTCAGGGTGG
TCCGCTGGAGCATGTGATCGCGGGCAAAGCCGTGGCGTTCTTTGAGGCACTGCAACCGGAGTTCAAAGAATATAGCCGTTTGGTCGTGG
AGAATGCGAAGCGTCTGGCCGAGGAACTGGCGCGTCGTGGTTACCGCATTGTGACCGGTGGCACGGATAACCACTTGTTTCTGGTCGAT
CTGCGCCCGAAAGGCCTGACCGGTAAAGAGGCTGAGGAACGCTTGGACGCGGTGGGCATCACCGTTAACAAAAATGCGATTCCGTTTGA
CCCGAAGCCTCCGCGTGTGACTAGCGGTATTCGCATCGGTACCCCTGCTATCACTACCCGCGGTTTCACGCCGGAAGAAATGCCGCTGG
TTGCGGAATTAATCGATCGCGCGCTGCTGGAAGGTCCGAGCGAAGCCCTGCGTGAAGAGGTCCGCCGTCTGGCACTGGCACACCCGATG
CCGTAA

b. Translated protein sequence. The TEV protease cleavage sequence is highlighted in green. The *Tth*SHMT sequence starts from Ser3 after the expression tag is cleaved.

MAHHHHHHVGTGSNDDDDKSTSLYKKAGSAAAPFTENLYFQSTLKRDEALFELIALEEKRQREGLELIASENFVSKQVREAVGSVLTNK
YAEGYPGARYYGGCEVIDRVESLAIERAKALFGAAWANVQPHSGSQANMAVYMALMEPGDTLMGMDLAAGGHLTHGSRVNFSGKLYKVV
SYGVRPDTELIDLEEVRRLALEHRPKVIVAGASAYPRFWDFKAFREIADEVGAYLVVDMAHFAGLVAAGLHPNPLPYAHVVTSTTHKTL
RGPRGGLILSNDPELGKRIDKLIFPGIQGGPLEHVIAGKAVAFFEALQPEFKEYSRLVVENAKRLAEELARRGYRIVTGGTDNHLFLVD
LRPKGLTGKEAEERLDAVGITVNKNAIPFDPKPPRVTSGIRIGTPAITTRGFTPEEMPLVAELIDRALLEGPSEALREEVRRLALAHPM
P

Figure S9. Sequences for SHMT from *Thermus thermophilus*.

(**A**) Codon-optimized DNA sequence.

ATGGCACATCATCACCATCACCACGTGGGCACCGGTAGCAATGACGATGACGACAAGTCTACCAGCCTGTACAAAAAAGCCGGCTCCGC
GGCAGCGCCGTTCACCGAGAACTTGTACTTCCAGGGCGAAGCGAACCGCGGTTGGACTGGTCAGGAAAGCCTGAGCGACAGCGACCCAG
AGATGTGGGAACTGCTGCAGCGCGAGAAAGATCGCCAGTGTCGTGGTCTGGAACTGATCGCGTCGGAGAACTTTTGCAGCCGCGCTGCG
TTAGAGGCCCTGGGTAGCTGTCTGAATAACAAGTATTCTGAGGGCTATCCGGGCAAGCGTTACTACGGTGGCGCCGAAGTTGTTGACGA
AATTGAGCTGCTGTGCCAACGCCGTGCGCTGGAAGCGTTTGATCTGGACCCGGCACAGTGGGGTGTCAATGTCCAACCGTACAGCGGCA
GCCCTGCAAACCTGGCCGTGTATACGGCATTGCTGCAGCCTCACGACCGTATTATGGGCCTGGATCTGCCGGATGGCGGTCACCTGACG
CACGGTTACATGAGCGACGTGAAGCGTATCAGCGCGACGAGCATCTTTTTCGAGAGCATGCCGTACAAACTGAACCCAAAGACCGGCTT
GATTGACTATAATCAACTGGCGCTGACTGCTCGTCTGTTTCGCCCGCGCCTGATCATTGCCGGTACCTCCGCGTATGCCCGTCTGATTG
ATTATGCGCGTATGCGCGAAGTCTGCGACGAAGTTAAAGCGCATCTGCTTGCAGATATGGCGCACATTTCAGGCCTGGTTGCGGCGAAA
GTAATCCCGAGCCCGTTTAAACATGCCGATATCGTCACCACCACCCACAAGACCCTGAGAGGCGCACGTAGCGGTTTGATCTTTTA
TCGTAAGGGTGTGAAAGCCGTGGACCCGAAAACGGGTCGCGAGATCCCGTACACGTTCGAGGATCGTATTAATTTCGCAGTGTTTCCAA
GCTTGCAGGGTGGCCCGCATAACCACGCGATCGCGGCAGTTGCAGTTGCTCTGAAACAAGCGTGCACGCCGATGTTTCGTGAGTACAGC
CTGCAAGTCCTCAAAAACGCACGCGCGATGGCTGATGCTCTGCTGGAGCGTGGCTACTCTCTGGTGAGCGGTGGCACCGATAATCACCT
GGTGCTGGTCGATTTGCGTCCGAAGGGTCTGGATGGTGCCCGTGCCGAACGTGTCCTGGAACTGGTTAGCATCACGGCGAACAAGAATA
CGTGTCCGGGTGACCGTTCCGCAATTACCCCGGGTGGTTTGCGTTTGGGTGCACCGGCGTTAACCAGCCGTCAGTTTCGTGAAGATGAT
TTCCGTCGTGTGGTGGACTTTATTGACGAGGGTGTGAACATTGGTCTTGAAGTTAAGAGCAAGACCGCGAAGCTGCAAGACTTCAAAAG
CTTCCTGCTGAAAGACTCCGAAACCAGCCAACGTCTGGCGAATCTGCGCCAACGTGTTGAGCAATTCGCTCGCGCATTCCCGATGCCGG
GTTTCGATGAGCACTAATAA

(**B**) Translated protein sequence. The TEV protease cleavage sequence is highlighted in green. The hSHMT2 sequence starts from Gly37 after the expression tag is cleaved.

MAHHHHHHVGTGSNDDDDKSTSLYKKAGSAAAPFTENLYFQGEANRGWTGQESLSDSDPEMWELLQREKDRQCRGLELIASENFCSRAA
LEALGSCLNNKYSEGYPGKRYYGGAEVVDEIELLCQRRALEAFDLDPAQWGVNVQPYSGSPANLAVYTALLQPHDRIMGLDLPDGGHLT
HGYMSDVKRISATIFFESMPYKLNPKTGLIDYNQLALTARLFRPRLIIAGTSAYARLIDYARMREVCDEVKAHLLADMAHISGLVAAKV
IPSPFKHADIVTTTHKTLRGARSGLIFYRKGVKAVDPKTGREIPYTFEDRIFAVFPSLQGGPHNHAIAAVAVALKQACTPMFREYSLQ
VLKNARAMADALLERGYSLVSGGTDNHLVLVDLRPKGLDGARAERVLELVSITANKNTCPGDRSAITPGGLRLGAPALTSRQFREDDFR
RVV
DFIDEGVNIGLEVKSKTAKLQDFKSFLLKDSETSQRLANLRQRVEQFARAFPMPGFDEH

Figure S10. Sequences for SHMT from human mitochondria.
