# Supplementary material for: Revealing protonation states and tracking substrate in serine hydroxymethyltransferase with room-temperature X-ray and neutron crystallography
Source: Commun Chem. 2023 Aug 3;6:162. doi: 10.1038/s42004-023-00964-9 (PMC10397204; doi:10.1038/s42004-023-00964-9)
Supplement: Supplementary file 3 — Description of Additional Supplementary Files [file 42004_2023_964_MOESM3_ESM.pdf]

Description of Additional Supplementary Files

File name: Supplementary Data 1

Description: Room-temperature X-ray structure of hSHMT2

File name: Supplementary Data 2

Description: Room-temperature X-ray structure of TthSHMT/D-Ser complex

File name: Supplementary Data 3

Description: Room-temperature joint X-ray/neutron structure of TthSHMT/L-Ser complex

File name: Supplementary Data 4

Description: Room-temperature joint X-ray/neutron structure of TthSHMT
